# RF Spectrum Sensing Based on an Overdamped Nonlinear Oscillator Ring for Cognitive Radios

**DOI:** 10.3390/s16060844

**Published:** 2016-06-09

**Authors:** Zhi-Ling Tang, Si-Min Li, Li-Juan Yu

**Affiliations:** Guangxi Key Laboratory of Wireless Broadband Communication and Signal Processing, Guilin 541004, China; siminl@guet.edu.cn (S.-M.L.); yulj@guet.edu.cn (L.-J.Y.)

**Keywords:** spectrum sensing, nonlinear system, duffing oscillator, synchronization

## Abstract

Existing spectrum-sensing techniques for cognitive radios require an analog-to-digital converter (ADC) to work at high dynamic range and a high sampling rate, resulting in high cost. Therefore, in this paper, a spectrum-sensing method based on a unidirectionally coupled, overdamped nonlinear oscillator ring is proposed. First, the numerical model of such a system is established based on the circuit of the nonlinear oscillator. Through numerical analysis of the model, the critical condition of the system’s starting oscillation is determined, and the simulation results of the system’s response to Gaussian white noise and periodic signal are presented. The results show that once the radio signal is input into the system, it starts oscillating when in the critical region, and the oscillating frequency of each element is *f_o_*/*N*, where *f_o_* is the frequency of the radio signal and *N* is the number of elements in the ring. The oscillation indicates that the spectrum resources at *f_o_* are occupied. At the same time, the sampling rate required for an ADC is reduced to the original value, 1/*N*. A prototypical circuit to verify the functionality of the system is designed, and the sensing bandwidth of the system is measured.

## 1. Introduction

In the crowded electromagnetic environment, high spectral efficiency, and optimal communication performance are achieved by a cognitive radio communication system that senses the spectrum hole, adopting artificial intelligence techniques to adaptively adjust the transmission power, carrier frequency, and modulation system parameters in real time, allowing the system to adapt to changes in the external environment [1]. In a cognitive radio communication system, spectrum sensing is an important component that refers to obtaining radio-spectrum usage information by a cognitive user through a variety of signal-detection and -processing means. From the point of view of the function layer of wireless networks, spectrum sensing mainly involves a physical and data-link layer. The physical layer mainly focuses on a variety of specific local-detection algorithms, and the link layer mainly on user collaboration and optimization of local sensing, collaboration sensing, and the sensing mechanism [2].

In recent years, many local-detection methods have been proposed, with energy detection being the most common. In the energy-detection method, the average energy of the signal sampling is compared with a threshold to determine whether the spectrum is used [3]. The realization of this method is simple, and does not require prior information of the primary user, but because of the uncertainty regarding the noise power, the energy cannot be effectively detected, and the sensing time is increased when the signal-to-noise ratio (SNR) is lower than a certain threshold [4,5]. The energy detector cannot distinguish the main user signal from the noise and other interference, which leads to a high false-alarm rate. In order to improve its performance, the power spectral density separation (PSC) method can effectively reduce the false-alarm rate by calculating the ratio of the sub-band power to the total bandwidth power [6]. On this basis, the bandwidth can be scanned by a tunable tracking filter, which can be used to extract the spectrum occupancy information of several specific sub-bands [7]. Another type of spectrum-detection method is based on signal characteristics, including cyclostationary features [8,9]. In these methods, the cyclic spectrum density is obtained by a fast Fourier transform (FFT) after sampling the cyclic autocorrelation function, and the peak value occurs when the spectrum is occupied. Compressed sensing can also be used to obtain a flag bit to detect the occupancy of a spectrum according to the symbol-bit information [10]. In a multiple-antenna system, the characteristic value of the array’s signal correlation matrix can be used to detect the frequency spectrum [11,12]. In the case of unknown noise, power, and location of the main user information, the blind estimation of the spectrum can be carried out using the moment feature [13].

In view of the problem that the local-detection method is not reliable in the cases of shadow and deep fading, cooperative spectrum sensing among users in the link layer is needed [14]. This method is the key to optimizing the merging method to obtain the sensing result because it is needed to integrate the sensing results among multiple cognitive users. For the weighted combination method, the frog-leaping algorithm can be used to obtain optimal weights to improve the probability of correct detection [15,16]. In order to reduce the network load of cooperative spectrum sensing, the double-threshold cooperative spectrum-sensing algorithm based on trust has better flexibility [17]. In order to overcome the influence of channel fading, the adaptive global optimization algorithm is proposed to determine the relay node set, which solves the problem of performance degradation induced by redundant relay interference, the detection threshold of nonoptimal designs, channel transmission error rate, and other factors [18].

Although cooperative spectrum sensing is able to compensate for the lack of local-detection methods to a certain degree, it is necessary to shorten the detection time and reduce the false-alarm rate to improve the single-cognitive-user spectrum-sensing ability, taking into account the network latency and traffic load and the algorithmic complexity. In general, spectrum sensing firstly requires sampling the RF signals according to Nyquist’s law. With the continuous increase of the frequency of the carrier signal, an ADC’s sampling rate must also increase. Thus, its resolution and dynamic range will become worse, which will lead to a decline in spectrum-detection performance, or an increase in the cost of ADC operation under the same conditions [19].

In this paper, a spectrum-sensing method based on a unidirectionally coupled, overdamped nonlinear oscillator ring is proposed. First, the weak signal detection by a nonlinear oscillator is a type of time-domain signal-processing technology with stronger detection ability than the previous spectral method, high-order statistics, *etc.* [20,21,22]. Secondly, the nonlinear oscillator has an active self-tuning capability that can be synchronized with an external periodic driving signal under specific conditions. In addition, the circuit of the nonlinear oscillator is relatively simple, which can, in turn, simplify the structure of the cognitive radio system. In this paper, we discuss the theory of the structure of a nonlinear oscillator-ring system and the critical conditions of the system operating as a spectrum detector.

## 2. Basic Structure of a Coupled, Overdamped Duffing Oscillator Ring

**Figure 1:**
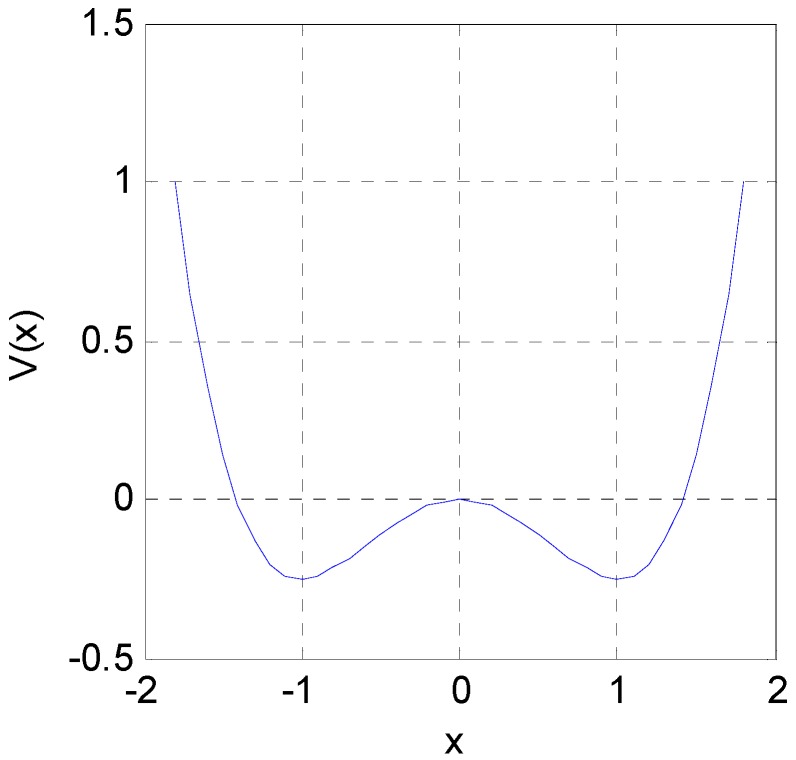
Potential function of a bistable system, showing that the motion can quickly converge to one of the two equilibrium points when the external force is missing.

A Duffing oscillator is a type of nonlinear oscillator that can be expressed as a nonlinear, two-order differential equation,

(1) $$\ddot{x}+\delta \dot{x}-\alpha x+\beta x^{3}=\gamma \eta (t)\;(\delta \geq 0)$$

where δ controls the size of the damping, α controls the size of the stiffness, β controls the nonlinearity of the restoring force, γ controls the amplitude of the external driving force, and η(*t*) indicates the external driving force. Moving the left-hand-side partial items in Equation (1) to the right-hand side, we obtain

(2) $$\begin{array}{l} \ddot{x}=-\delta \dot{x}+\alpha x-\beta x^{3}+\gamma \eta (t)\\ \;=\;-\delta \dot{x}-\dot{U}(x)+\gamma \eta (t)\;(\delta \geq 0) \end{array}$$

where $\ddot{x}$ is the inertial force, which can be ignored in the overdamped case. Then Equation (2) is rewritten as

(3) $$\begin{array}{l} \delta \dot{x}=\;-\dot{U}(x)+\gamma \eta (t)\;\\ \;=\;\alpha x-\beta x^{3}+\gamma \eta (t) \end{array}$$

which is called the Langevin equation. When α > 1, *U*(*x*) = −α*x*^2^/2 + β*x*^4^/4 is a bistable state potential function, which is used to describe the motion of a unit mass particle in a potential well [23]. Assuming that β = 1, α = 1, γ = 1, $x_{s1}=1$ and $x_{s2}=-1$ are the two equilibrium points, $x_{un}=0$ is the nonequilibrium point. As shown in Figure 1, the relationship between the bistable state potential and *x* shows that the motion converges quickly to one of the two equilibrium points when the external force is missing. Or to the general β and α, the equilibrium points are $x_{s}=\pm \sqrt{\alpha /\beta }$ and the barrier height at $x_{un}=0$ is ∆*U* = α^2^/4β.

**Figure 2:**
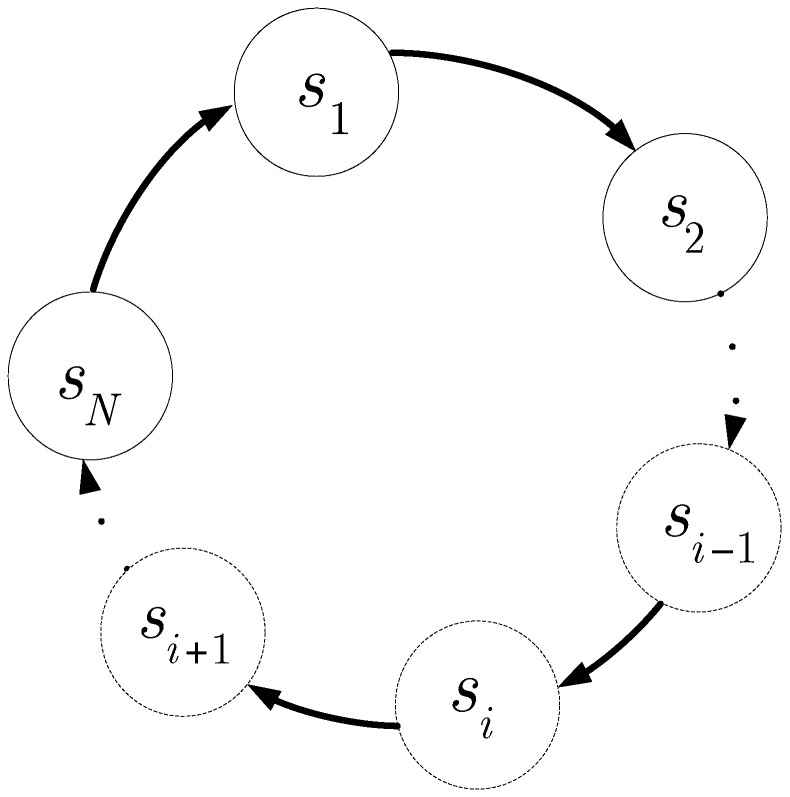
Structure of the unidirectionally coupled, overdamped nonlinear oscillator ring.

If the nonlinear oscillator is a unidirectionally coupled ring as shown in Figure 2, and the number of oscillators in the ring is *N*, the coupled bistable oscillator in the ring is expressed as follows:

(4) $$\delta {\dot{x}}_{i}=\alpha x_{i}-\beta x_{i}^{3}+k(x_{i-1}-x_{i})$$

where k is the linear coupling coefficient and i=1,2,…,N. For spectrum sensing of a cognitive radio, the coupled oscillator ring should not oscillate until it senses a radio signal from the antenna. The oscillation of the ring indicates that the radio-frequency spectrum has been occupied. Thus, the critical point at which the system starts oscillating must be defined. Letting the radio signal received by the antenna be *r*(*t*) = *a*(*t*)cos[ω*_c_* + θ(*t*)], where *a*(*t*) is the signal amplitude, θ(*t*) is the signal phase, ω*_c_* is the signal frequency, the radio signal is input to each element, Equation (4) is rewritten as

(5) $$\delta {\dot{x}}_{i}=\alpha x_{i}-\beta x_{i}^{3}+k(x_{i-1}-x_{i})+r(t)$$

## 3. Dynamics and Analysis

### 3.1. Dynamic Model

**Figure 3:**
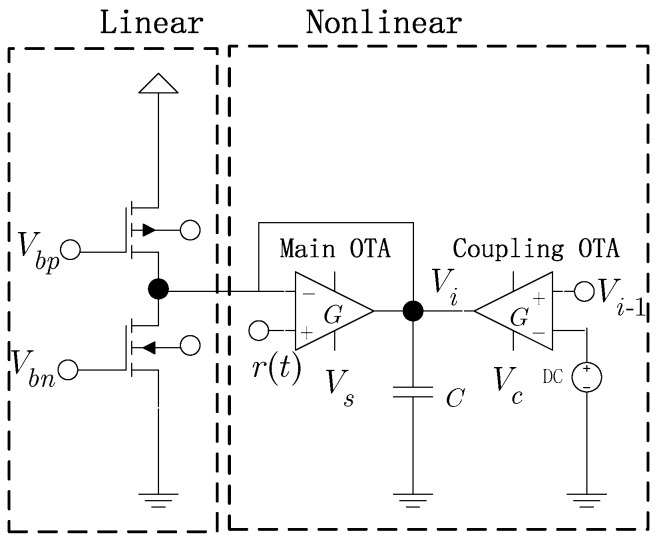
Circuit of an overdamped nonlinear oscillator, which can be an element of a unidirectionally coupled, overdamped nonlinear oscillator ring.

The practical circuit of a bistable, overdamped, nonlinear oscillator as shown in Figure 3 has been used to detect weak signals [24]. The circuit can be divided into two parts: one a linear part composed of two field-effect transistors (FETs), and the other a nonlinear part comprised of two transconductance operational amplifiers. A model for describing the nonlinear phenomena of this circuit is defined as

(6) $$C{\dot{V}}_{i}=-gV_{i}+I_{s}\tanh[c_{s}(V_{i}-r(t))]+I_{c}\tanh[c_{c}(V_{dc}-V_{i-1})]$$

where *C* is load capacitance; $gV_{i}=I_{sc}-I_{o}$, where $I_{o}$ is the sum of the steady-state current in both linear and nonlinear currents and $I_{sc}=I_{p}-I_{n}$ is a linear part of the effective current in the saturation state of the transistor; $I_{p}$ and $I_{n}$ are the leakage currents through the N-channel and the P-channel FET; $V_{i}$ is the oscillator’s output and $V_{i-1}$ is the output of the previous oscillator; $c_{s}=\sqrt{\eta /I_{s}}$, $c_{c}=\sqrt{\eta /I_{c}}$, and β are the process parameters; $I_{s}$ and $I_{c}$ are the main operational transconductance amplifier (OTA) bias current and the coupled OTA bias current, respectively; and r(t) is the signal to be detected. Equation (6) is a variation of Equation (5), which also has the characteristics of an overdamped bistable state.

**Figure 4:**
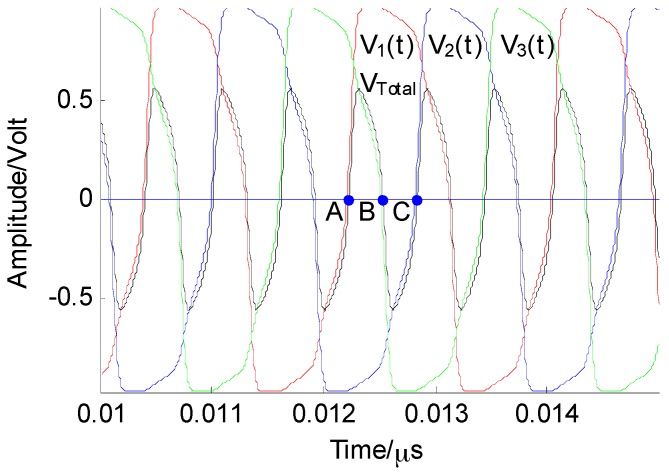
Numerical-simulation oscillation waveform of the system when N=3, $c_{s}=c_{c}=1$, C=0.1 pF, g=1/1000 Ω, r(t)=0 V, $V_{dc\;}=0\text{ V}$, $I_{s}=120\;\mu A$, and $I_{c}=100\;\mu A$.

In order to form a ring as shown in Figure 2, the output of the previous element is coupled into the element by the coupling OTA shown in Figure 3, and the signal itself is coupled to the next element. The generation of the oscillation is related to *N*, the number of elements in the ring. When *N* has an even value, the system is in a stable state; the system is not stable when *N* has an odd value [25]. For the circuit shown in Figure 3, *C*, $I_{o}$, $I_{sc}$, $I_{s}$, and $I_{c}$ must be adjusted to the appropriate values to generate the periodic signal. Via Equation (6), $I_{s}$ is defined as the nonlinear coefficient representing the bistability of the circuit, and $I_{c}$ is the coupling coefficient among the nonlinear oscillators. $c_{s}$ and $c_{c}$ are constants during signal processing. Provided N=3, $c_{s}=c_{c}=1$, C=0.1 pF, g=1/1000 Ω, r(t)=0 V, $V_{dc}=0\text{ V}$, $I_{s}=120\;\mu A$ and $I_{c}=100\;\mu A$, and substituting into the following system dynamics equations:

(7) $$\{\begin{array}{c} C{\dot{V}}_{1}=-gV_{1}+I_{s}\tanh(V_{1})-I_{c}\tanh(V_{3})\;\\ C{\dot{V}}_{2}=-gV_{2}+I_{s}\tanh(V_{2})-I_{c}\tanh(V_{1})\;\\ C{\dot{V}}_{3}=-gV_{3}+I_{s}\tanh(V_{3})-I_{c}\tanh(V_{2})\; \end{array}$$

where the numerical-simulation waveform of each oscillator is shown in Figure 4. From Figure 4, it can be seen that while the frequency of each oscillator is the same, the phase difference between them is 2π/3. Next, the critical point at which the system starts oscillating, as shown in Figure 4, must be discussed, including the effect of the coupling and nonlinear coefficients. This will provide enough information for us to control the ring system for spectrum-sensing applications.

### 3.2. Relationship between Oscillation Frequency and Currents (I_c_,I_s_)

For spectrum-sensing applications, it is a prerequisite for the unidirectionally coupled oscillator to generate periodic oscillation; Therefore, its state-transition condition is critical. For this purpose, the fixed points of the system are analyzed according to Equation (6), and the bifurcation points are determined with the change of the coupling and nonlinear coefficients. Letting N=3, r(t)=0 V, $V_{dc}=0\text{ V}$, $\bar{g}=-g/C$, ${\bar{I}}_{s}=I_{s}/C$ and ${\bar{I}}_{c}=I_{c}/C$, the system can be represented as

(8) $$\{\begin{array}{c} {\dot{V}}_{1}=-\bar{g}V_{1}+{\bar{I}}_{s}\tanh(V_{1})-{\bar{I}}_{c}\tanh(V_{3})\;\\ {\dot{V}}_{2}=-\bar{g}V_{2}+{\bar{I}}_{s}\tanh(V_{2})-{\bar{I}}_{c}\tanh(V_{1})\\ {\dot{V}}_{3}=-\bar{g}V_{3}+{\bar{I}}_{s}\tanh(V_{3})-{\bar{I}}_{c}\tanh(V_{2}) \end{array}$$

By linearization or the Hartman-Grobman theory, the stability of the fixed point of the dynamical system can be determined by the Jacobian eigenvalues and eigenvectors of the fixed point. If the eigenvalues have positive real parts, the fixed points along the feature vectors are unstable; if the eigenvalues have negative real parts, the fixed points along the feature vectors are stable [26]. The coupled system is rewritten in a more compact form as: $dx_{i}/dt=f(x_{i},x_{i-1},\bar{g},{\bar{I}}_{c},{\bar{I}}_{s})$, i=1,…,N. For the coupled system in which *N* = 3, the Jacobian at the origin, $\left(x_{1},x_{2},x_{3})=(0,0,0\right)$, is

(9) $$J=(df)=\left(\begin{array}{ccc} -g+{\bar{I}}_{s} & 0 & -{\bar{I}}_{c}\\ -{\bar{I}}_{c} & -g+{\bar{I}}_{s} & 0\\ 0 & -{\bar{I}}_{c} & -g+{\bar{I}}_{s} \end{array}\right)$$

**Figure 5:**
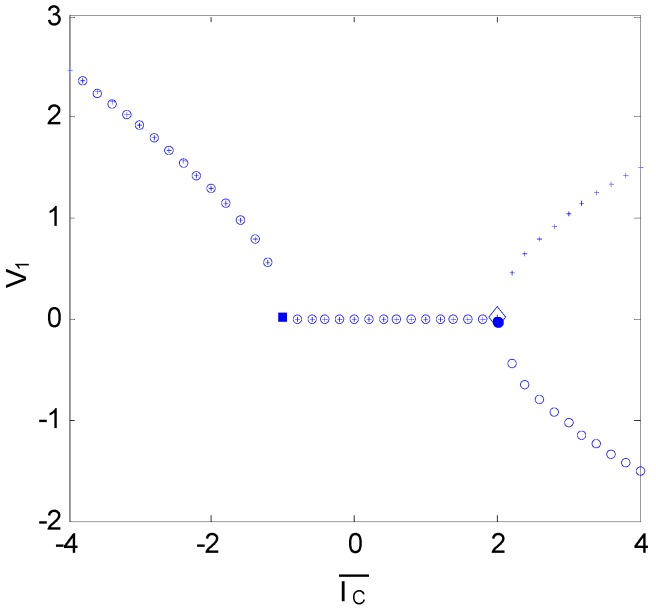
Bifurcation diagram of the system (N=3, $\bar{g}=2$, ${\bar{I}}_{s}=1$). Once the unstable bifurcation point is reached, the system begins to oscillate. Solid dots indicate the unstable bifurcation point.

Letting $-\bar{g}+{\bar{I}}_{s}={\bar{I}}_{gs}$, the Jacobian eigenvalues are $\lambda_{1}={\bar{I}}_{gs}+{\bar{I}}_{c}$, $\lambda_{2,3}={\bar{I}}_{gs}-{\bar{I}}_{c}/2\pm i\left(\sqrt{3}/2\right)I_{c}$. From the eigenvalues, we find that there are two local bifurcation points apart from the origin: one is the bifurcation of the steady state at ${\bar{I}}_{c}=-{\bar{I}}_{gs}$, and the other is the Hopf bifurcation at ${\bar{I}}_{c}=2{\bar{I}}_{gs}$. The bifurcation diagram of the system is shown in Figure 5, which shows the steady-state bifurcation point at ${\bar{I}}_{c}=-1$; note that a pitch bifurcation occurs at ${\bar{I}}_{c}=2$, becoming the two branches of the unstable nontrivial equilibrium points. Once the unstable bifurcation point ${\bar{I}}_{c}^{*}$ is reached, the system begins to oscillate. When $\bar{g}=2$ and ${\bar{I}}_{s}=1$, ${\bar{I}}_{c}^{*}=2$.

The critical coupling coefficient and the frequency of the system oscillation can be determined according to Equation (8). Although the oscillation frequency of the system can be roughly estimated from Figure 4, an accurate computation of the oscillation period can be obtained based on the decoupling method. As shown in Figure 1, the main time of a particle moving from the left (negative) to the right (positive) is the period from the negative state across the potential barrier, while the time “rolled” to positive-state time over the potential barrier is negligible. Figure 4 shows that the rest of the elements are approximately in a steady state when an element climbs over a potential barrier. Therefore, the system can be decoupled in the calculation of the cycle of a single element, and the coupled term is regarded as a constant. The calculation of the oscillation period is divided into two parts; that is, the transition from the positive state to the negative state and vice versa. Assuming that the element 1 locates at the positive minimum at *t* = 0, the time evolution from the positive state to the negative state can be obtained from the following integral:

(10) $$t_{1}=\int_{V_{1+}}^{0}\frac{dV_{1}}{f_{1}(V_{1})}$$

where $f_{1}(V_{1})=-\bar{g}V_{1}+{\bar{I}}_{s}\tanh(V_{1})-{\bar{I}}_{c}\tanh(V_{3+})$, and $V_{\text{1+}}$ and $V_{3+}$ are the positive minima of elements 1 and 3, respectively. Letting $h_{1}(V_{1})=-f_{1}(V_{1})$, Equation (10) is rewritten as

(11) $$t_{1}=\int_{0}^{V_{1+}}\frac{dV_{1}}{h_{1}(V_{1})}$$

Because $h_{1}(V_{1})$ has a sharp peak at the inflection point, $V_{1m}=\text{asech(}1/\sqrt{{\bar{I}}_{s}/g})$, and $h_{1}(V_{1})$ is expanded at $V_{1m}$ as

(12) $$\begin{array}{l} h_{1}(V_{1})≃h_{1}(V_{1m})+\frac{{\left(V_{1}-V_{1m}\right)}^{2}}{2}h_{1}^{\prime \prime }(V_{1m})\\ \;≃A_{1}V_{1}^{2}+B_{1}V_{1}+C_{1} \end{array}$$

where $A_{1}=h_{1}^{\prime \prime }(V_{1m})/2$, $B_{1}=-V_{1m}h_{1}^{\prime \prime }(V_{1m})$, and $C_{1}=h_{1}(V_{1m})+V_{1m}^{2}h_{1}^{\prime \prime }(V_{1m})/2$. Then,

(13) $$t_{1}≃\int_{0}^{V_{1+}}\frac{dV_{1}}{A_{1}V_{1}^{2}+B_{1}V_{1}+C_{1}}$$

When the integral limit→∞, then

(14) $$t_{1}≃\frac{2}{D_{1}}[\frac{\pi }{2}-\text{atan}(\frac{B_{1}}{D_{1}})]$$

where $D_{1}=\sqrt{4A_{1}C_{1}-B_{1}^{2}}$. According to the same principle, another part of the oscillation period is obtained by calculating the transition of element 2 from the negative state to the positive state:

(15) $$t_{2}=\int_{V_{2-}}^{0}\frac{dV_{2}}{f_{2}(V_{2})}$$

where $f_{2}(V_{2})=-\bar{g}V_{2}+{\bar{I}}_{s}\tanh(V_{2})-{\bar{I}}_{c}\tanh(V_{1-})$, and $V_{2-}$ and $V_{1-}$ are the negative minima of elements 2 and 1, respectively. The evaluation result for the integral is

(16) $$t_{2}≃\frac{2}{D_{2}}[\frac{\pi }{2}-\text{atan(}\frac{B_{2}}{D_{2}})]$$

where $B_{2}=-V_{2m}h_{2}^{\prime \prime }(V_{2m})$, $C_{2}=h_{2}(V_{2m})+V_{2m}^{2}h_{2}^{\prime \prime }(V_{2m})/2$, $D_{2}=\sqrt{4A_{2}C_{2}-B_{2}^{2}}$, and $h_{2}(V_{2})=-f_{2}(V_{2})$. In the end, the period of the superposition of the three elements’ oscillation signal is $T_{\sum }=t_{1}+t_{2}$. The corresponding frequency is

(17) $$f_{\text{ring}}=\frac{1}{T_{\sum }}=\frac{1}{t_{1}+t_{2}}$$

In order to solve the critical point of the system oscillation, the potential function of Equation (6) is expressed as

(18) $$U=-\int_{0}^{V_{1}}f(V_{1})dV_{1}$$

Using Equation (17) to find the critical coupling point, and letting $f(V_{1})=0$ and $f\prime (V_{1})=0$ at the point $V_{1m}$, we obtain

(19) $$\bar{g}V_{1}-{\bar{I}}_{s}\tanh(V_{1m})+{\bar{I}}_{cc}\tanh(V_{3+})\;=\;0$$

and

(20) $$\bar{g}V_{1}-{\bar{I}}_{s}\tanh(V_{1m})+{\bar{I}}_{cc}\tanh(V_{3+})\;=\;0$$

**Figure 6:**
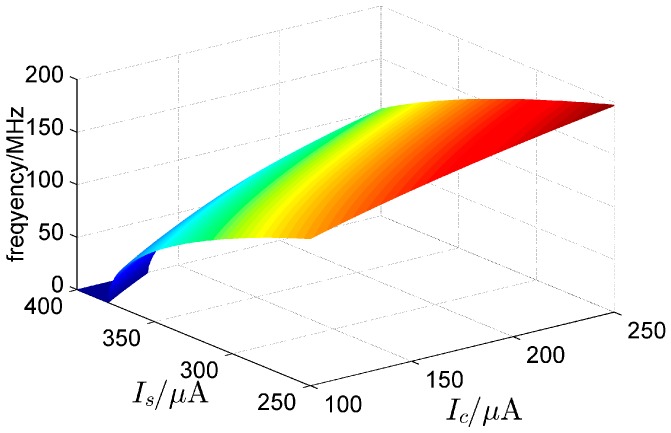
Variation of the oscillation frequency with currents ($I_{c}$, $I_{s}$), which indicates that the oscillation frequency of the system can be determined by ($I_{c}$, $I_{s}$) (N=3, $c_{s}=c_{c}=1$, C=0.1 pF, g=1/1000 Ω, r(t)=0 V and $V_{dc}=0\text{ V}$).

Solving $I_{cc}$, we obtain

(21) $$\begin{array}{l} {\bar{I}}_{cc}=\left[-\bar{g}V_{1m}+{\bar{I}}_{s}\tanh(V_{1m})\right]\coth(V_{3+})\\ \;\approx -\bar{g}V_{1m}+{\bar{I}}_{s}\tanh(V_{1m})\\ \;\approx -\bar{g}a\mathrm{sech}\left[\sqrt{\frac{g}{{\bar{I}}_{s}}}\right]+{\bar{I}}_{s}\tanh\left[a\mathrm{sech}\left[\sqrt{\frac{g}{{\bar{I}}_{s}}}\right]\right] \end{array}$$

which describes the relationship between the critical coupling coefficient ${\bar{I}}_{cc}$ and the critical nonlinear coefficient ${\bar{I}}_{s}$. According to Equations (17) and (21), the variation of the oscillation frequency with currents ($I_{c}$, $I_{s}$) is shown in Figure 6. It shows that the oscillation frequency of the system increases with the increase of $I_{c}$, and decreases with the increase of $I_{s}$.

### 3.3. Spectrum Sensing

Although we have shown that the unidirectionally coupled, overdamped nonlinear oscillator ring can generate an oscillation signal once the coupling coefficient (current) exceeds the critical coupling point, it cannot be said, however, that it can sense the RF spectrum. Only when the output of the antenna is fed to the system, and the frequency of the system can be locked to the frequency of the external RF signal, can the occupancy of the spectrum be recognized. In addition, the influence of the noise in the spectrum-sensing channel creates a factor of uncertainty.

Assuming only the Gaussian white noise $\sqrt{2D}\xi (t)$, output from the antenna is considered, and the noise is a random process with a variance of D and a mean of 0. Letting $r(t)=\sqrt{2D}\xi (t)$, Equation (6) is rewritten as

(22) $$C{\dot{V}}_{i}=-gV_{i}+I_{s}\tanh[c_{s}(V_{i}-\sqrt{2D}\xi (t))]+I_{c}\tanh[c_{c}(V_{dc}-V_{i-1})]$$

If the Euler forward integration method is used for the numerical analysis of differential equations, under the conditions $c_{s}=c_{c}=1$, $V_{dc}=0\text{ V}$, $\bar{g}=-g/C$, ${\bar{I}}_{s}=I_{s}/C$ and ${\bar{I}}_{c}=I_{c}/C$, Equation (22) is changed into

(23) $$V_{i}(t+\Delta t)=V_{i}(t)+\Delta t\left[-\bar{g}V_{i}(t)+{\bar{I}}_{s}\tanh[V_{i}(t)-\sqrt{2D}\xi (t)]-{\bar{I}}_{c}\tanh[V_{i-1}(t)]\right]$$

**Figure 7:**
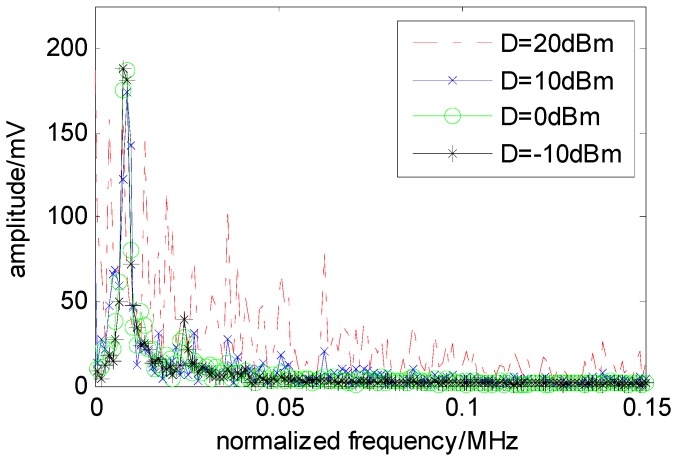
Normalized frequency spectrum of system oscillation waveform corresponding to different Gaussian white-noise variances.

Through numerical simulation in MATLAB, the bifurcation characteristics of the system with Gaussian white noise are obtained under different noise variances. Table 1 lists the different critical coupling points for different noise variances and shows that the variation of the critical coupling point is minor when Gaussian white noise is fed into the system. Figure 7 shows the spectrum of the system oscillation waveform when the noise variances are 20, 10, 0, −10, −20 and −30 dBm. Except for the variance of 20 dBm, the frequency of the oscillation waveform is the same for all of the other cases.

Next, assuming that only the RF signal output from the antenna is considered, spectrum sensing requires a radio signal to be fed into the system. We know from Figure 6 that different critical currents $\left(I_{sc},I_{cc}\right)$ arise that put the system into a critical state on the verge of oscillation. When the RF signal, r(t)=A(t)cos[ωt+ϕ(t)], is fed into the system as in Equation (6), where A(t) is the instantaneous amplitude, ϕ(t) is the instantaneous phase, and ω is the carrier frequency, Equation (6) is rewritten as

(24) $${\dot{V}}_{i}=-\bar{g}V_{i}+{\bar{I}}_{s}\tanh\{V_{i}-A(t)\cos[\omega t+\phi (t)]\}-{\bar{I}}_{c}\tanh(V_{i-1})$$

**Figure 8:**
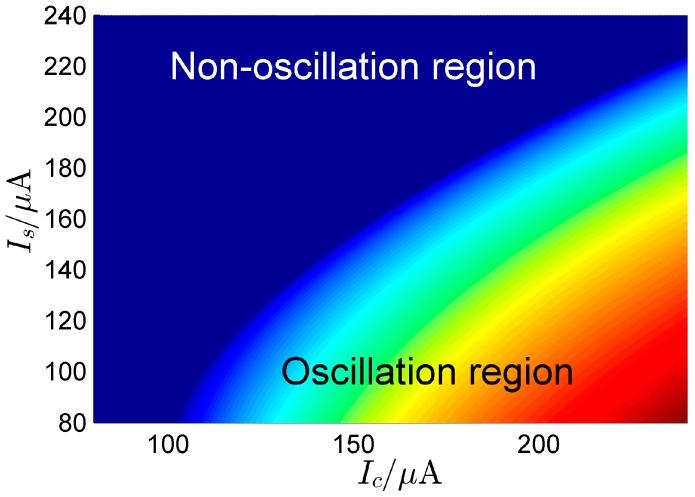
Oscillation and non-oscillation regions related to ($I_{c}$, $I_{s}$) (N=3, $c_{s}=c_{c}=1$, C=0.1 pF, g=1/1000 Ω, $V_{dc}=0\text{ V}$).

**Figure 9:**
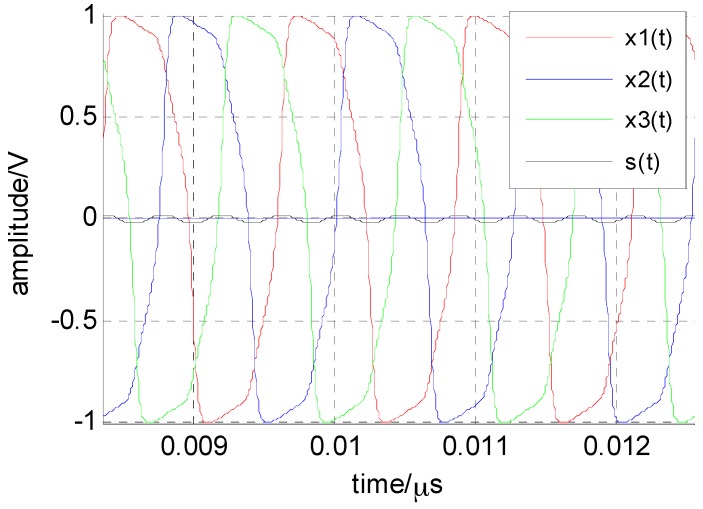
When the external radio signal with frequency $f_{s}$ is fed into the system, the frequency of the oscillation waveform of each element is locked to $f_{s}/3$ (N=3, $c_{s}=c_{c}=1$, C=0.1 pF, g=1/1000 Ω, $V_{dc}=0\text{ V}$, $I_{s}=220\;\mu A$, $I_{c}=300\;\mu A$, $f_{s}=2.\text{421 GHz}$).

The system represented by Equation (24) is a nonautonomous, or forced oscillation, system. According to the theory of nonlinear oscillators, when the difference between the frequency of the external signal and the free oscillation frequency of the nonlinear oscillator is small enough, then the frequency of the oscillation will be locked to the external signal. Therefore, such a system functions as a spectrum-sensing device, operating in the critical region of the oscillation when no signal is present. Figure 8 shows the oscillation and non-oscillation regions related to (*I_C_*, *I_S_*). There is a boundary between the two regions that is the critical transition from the non-oscillation state to the oscillation state. When there is no signal, the system is in a non-oscillation region; when the antenna output contains the signal, the system will cross the critical point into the oscillation region, according to Equation (24) and Figure 8. Then, each element in the system will oscillate as shown in Figure 4. Considering a radio signal to be detected, *s*(*t*), with a carrier frequency of 2.421 GHz and a power of −50 dBm, at first the currents is set as (*I_S_* = 220 μA, *I_C_* = 300 μA); thus the system is in the critical region and there is no oscillation waveform. When the radio signal is fed into the system, the output waveform of each element is shown in Figure 9, which shows that the frequency of the oscillation waveform {*x*_1_(*t*), *x*_2_(*t*), *x*_3_(*t*)} is locked to the carrier signal *s*(*t*), and its amplitude is far greater than the signal.

### 3.4. Circuit Experiments

**Figure 10:**
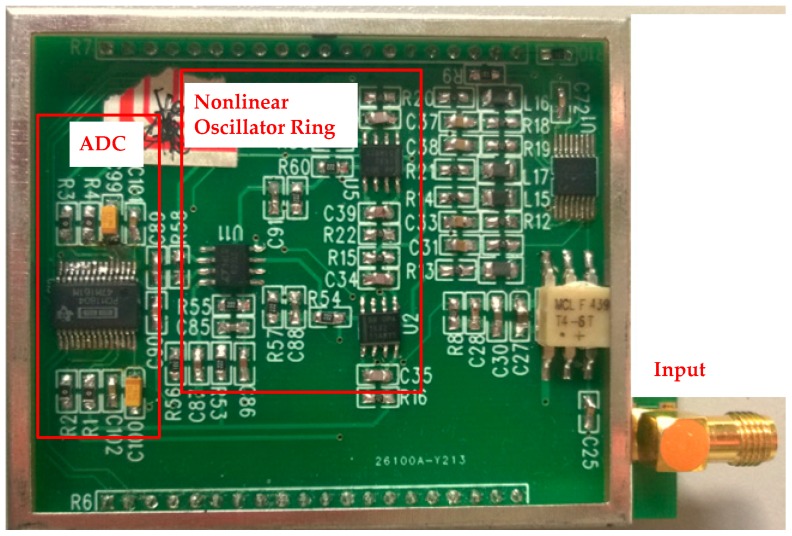
Experimental spectrum-sensing circuit based on a unidirectionally coupled, overdamped nonlinear oscillator.

Based on the circuit and the structure of the unidirectionally coupled, overdamped nonlinear oscillator shown in Figure 2, we designed an experimental spectrum-sensing circuit composed of three elements. The circuit, which includes a nonlinear oscillator ring, and an ADC, is shown in Figure 10.

**Figure 11:**
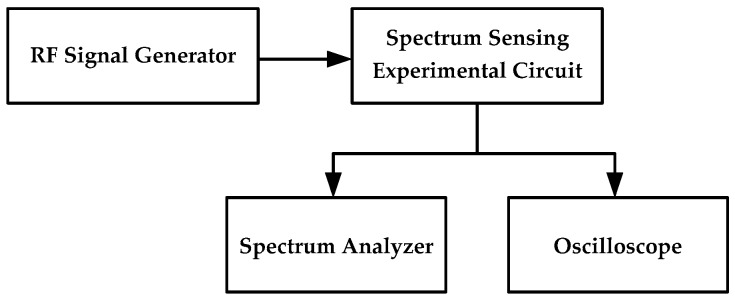
Setup for spectrum-sensing experiment.

**Figure 12:**
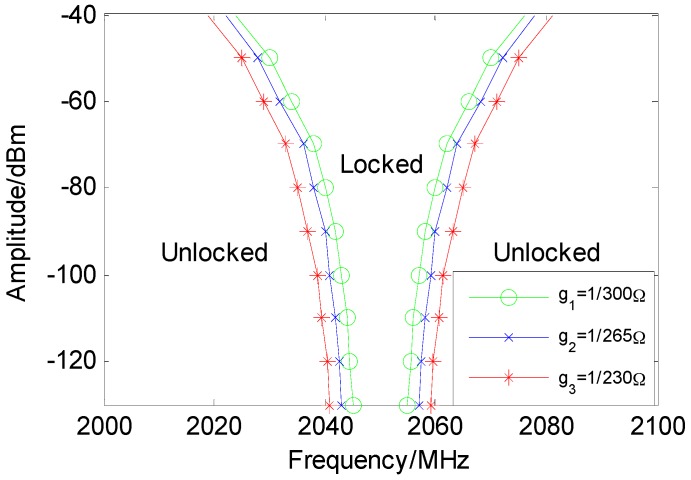
Relationship between the spectrum-sensing range of the system and the amplitude of the input signal intensity, or the conductivity, g($I_{sc}=220\;\mu A$, $I_{cc}=300\;\mu A$).

The setup for the spectrum-sensing experiment is shown in Figure 11. First, the critical point (*I_SC_*, *I_CC_*) is adjusted to make the circuit system work in the critical region of oscillation. Next, the signal generator is used to generate the RF signal that is fed to the circuit system. When the frequency of the signal falls into the spectrum-sensing range, the circuit system starts to oscillate; the frequency of the oscillation signal is 1/3 of the RF signal. As shown in Figure 12, by changing the intensity and frequency of the input signal, the relationship between the spectrum-sensing bandwidth and the amplitude of the input signal, or the conductivity, *g*, can be obtained by observing whether the circuit oscillates. In Figure 12, the region labeled “unlocked” is where the system is not synchronized to the external RF signal, while the region labeled “locked” is where the system is synchronized to the external RF signal.

## 4. Discussion

Because the overdamped Duffing oscillator cannot oscillate by itself, the inertial term in the Duffing equation can be ignored, which simplifies the model analysis in this paper. The simplified model is a bistable system. When *N* overdamped bistable systems are unidirectionally coupled into a ring system, oscillation may occur under specific conditions. The circuit of this bistable system is shown in Figure 3 and is modeled by Equation (6). Through analysis, the oscillation frequency of the ring system is mainly determined by the current (*I_S_*, *I_C_*). The relationship between the free oscillation frequency and the current of the system is shown in Figure 6, and the response of the system is divided into oscillation and the non-oscillation regions, as shown in Figure 8. When the system is used for spectrum sensing, the system operates in the critical non-oscillation region. Once an RF signal appears in the channel, the system enters the oscillation region and its oscillation frequency is locked to the RF signal. The result obtained by numerical simulation is shown in Figure 9, which indicates that this phenomenon will occur as long as the current (*I_S_*, *I_C_*) is appropriate. The frequency of each element is locked to 1/*N* of the frequency of the external radio signal. This result provides two benefits for spectrum sensing: (1) weak radio signal detection is converted to a stronger oscillator waveform detection, which reduces the requirement of the ADC’s dynamic range; (2) the ADC’s sampling rate is reduced. In addition, regarding the Gaussian white noise in the radio channel, the data reported in Table 1 and Figure 7 show that it has no real effect on the oscillation of the system.

Through practical circuit experiments, spectrum-sensing functionality is verified. Because the frequency of the system is determined by the current (*I_S_*, *I_C_*), it is necessary to determine the critical current (*I_SC_*, *I_CC_*) according to its operating frequency band. Afterwards, the frequency of the system can be locked to external radio signals. From the experiments, the relationship between the spectrum-sensing range of the system and the amplitude of the input signal intensity, or the conductivity, *g*, are obtained. The data show that the spectrum-sensing bandwidth will increase with the growth in amplitude of the input signal intensity. In addition, the growth of *g* in Equation (6) will also expand the spectrum-sensing range. If a cognitive radio system needs to be aware of the band beyond that provided by a single-ring system, multiple-ring systems operating at different currents (*I_S_*, *I_C_*) are combined, with each system covering a specific frequency band. Thus, spectrum sensing along the entire frequency band can be achieved. Furthermore, a more complicated current control circuit is worth studying in order to extend the frequency band of a single ring system in the future.

The conventional method needs A/D sampling and complicated digital signal processing, which is time consuming. While the proposed scheme performs spectrum sensing at time domain, therefore the detection time until finding the existence of the primary signal is shorter, and the probability of interfering with the primary user is reduced.

## 5. Conclusions

In this paper, a spectrum-sensing method based on a unidirectionally coupled, overdamped nonlinear oscillator ring is discussed in detail. The ring system is composed of *N* overdamped Duffing oscillators, which is simplified to a bistable system. An overdamped Duffing oscillator can be realized by a simple circuit, which is easy to make into an integrated circuit. If it is unidirectionally coupled to a ring, the system will spontaneously generate oscillations related to the critical currents (*I_SC_*, *I_CC_*). The critical currents divide the response of the system into oscillation and non-oscillation regions. When the system operates in the critical non-oscillation region, it will not oscillate. However, once the external RF signals are fed into each element of the system, they start to oscillate and the frequency is locked to the RF signal. Even if the RF signal is weak, the system still exhibits this characteristic. Regarding the usual Gaussian white noise in radio channels, there is no obvious effect on the oscillation of the system. These features are not only utilized to achieve spectrum sensing, but they also reduce the requirements of the ADC’s dynamic range and sampling rate. The circuit experiments show that the spectrum-sensing bandwidth is related to the amplitude of the detected RF signal and the conductivity of the element. If multiple spectrum-sensing systems operating with different currents (*I_SC_*, *I_CC_*) are combined, so that each system covers a different frequency band, wider-bandwidth spectrum sensing can be achieved.

## Figures and Tables

**Table 1:** Critical coupling points of different noise variances (N=3, $c_{s}=c_{c}=1$, C=0.1 pF, g=1/1000 Ω, $V_{dc}=0\text{ V}$, $I_{s}=120\;\mu A$, $I_{c}=100\;\mu A$).

D (dBm)	20	10	0	−10	−20	−30
${\bar{I}}_{cc}$	2.213	2.061	2.043	2.039	2.035	2.032
